# Estimation of homeostatic dysregulation and frailty using biomarker variability: a principal component analysis of hemodialysis patients

**DOI:** 10.1038/s41598-020-66861-6

**Published:** 2020-06-25

**Authors:** Yuichi Nakazato, Tomoko Sugiyama, Rena Ohno, Hirofumi Shimoyama, Diana L. Leung, Alan A. Cohen, Riichi Kurane, Satoru Hirose, Akihisa Watanabe, Hiromi Shimoyama

**Affiliations:** 1 Division of Nephrology, Yuai Nisshin Clinic, Hakuyukai Medical Corporation, Saitama-City, Saitama Japan; 2 Department of Pathology, Yale University, New Haven, CT USA; 3 Groupe de recherche PRIMUS, Department of Family Medicine, University of Sherbrooke, Sherbrooke, Quebec Canada; 4 Division of Nephrology, Yuai Clinic, Hakuyukai Medical Corporation, Saitama-City, Saitama Japan; 5 Division of Nephrology, Yuai Mihashi Clinic, Hakuyukai Medical Corporation, Saitama-City, Saitama Japan; 6 Division of Nephrology, Yuai Nakagawa Clinic, Hakuyukai Medical Corporation, Saitama-City, Saitama Japan

**Keywords:** Ageing, Complexity, Molecular fluctuations, Regulatory networks, Renal replacement therapy, Diabetes, Geriatrics, Biomarkers

## Abstract

Increased intraindividual variability in several biological parameters is associated with aspects of frailty and may reflect impaired physiological regulation. As frailty involves a cumulative decline in multiple physiological systems, we aimed to estimate the overall regulatory capacity by applying a principal component analysis to such variability. The variability of 20 blood-based parameters was evaluated as the log-transformed coefficient of variation (LCV) for one year’s worth of data from 580 hemodialysis patients. All the LCVs were positively correlated with each other and shared common characteristics. In a principal component analysis of 19 LCVs, the first principal component (PC1) explained 27.7% of the total variance, and the PC1 score exhibited consistent correlations with diverse negative health indicators, including diabetes, hypoalbuminemia, hyponatremia, and relative hypocreatininemia. The relationship between the PC1 score and frailty was subsequently examined in a subset of the subjects. The PC1 score was associated with the prevalence of frailty and was an independent predictor for frailty (odds ratio per SD: 2.31, *P* = 0.01) using a multivariate logistic regression model, which showed good discrimination (c-statistic: 0.85). Therefore, the PC1 score represents principal information shared by biomarker variabilities and is a reasonable measure of homeostatic dysregulation and frailty.

## Introduction

Studies on variability in blood pressure, plasma glucose levels, hemoglobin concentration, and other parameters have commonly reported associations with adverse outcomes^1–3^. In our previous study examining patients receiving maintenance hemodialysis (HD), variability in many other blood-based laboratory parameters was also related to several adverse conditions, such as impaired mobility, hospital admission, increased mortality, and hypoalbuminemia^4^. These conditions are in fact elements of frailty, and frailty is considered to be a state of functional decline in many physiological systems^5^. Therefore, we speculated that the variability in laboratory parameters may reflect the dysfunction of corresponding regulatory systems and may also be a measure of frailty. Consistent with this idea, the variability of serum albumin concentrations increases with ageing, and this movement accelerates prior to death^6^. Others have also suggested a link between physiological regulation and variability in other biological variables^7–10^. However, the variability of a single parameter may not properly represent the *dysregulation across multiple physiological systems* in frailty^11^.

To develop a comprehensive measure of physiological dysregulation that is consistent with the concept of frailty, we applied a principal component analysis (PCA) to a set of variabilities in laboratory parameters. This procedure, performed on all study participants (n = 580), yielded principal component scores (PC scores) for each subject. The properties of the PCA model and its derived PC score were first investigated in this population. Following this, the relationship between the PC score and frailty was analyzed in a subpopulation of 109 subjects.

Our aims for this study were (1) to examine whether the PC1 score is an appropriate measure of physiological dysregulation and frailty, and (2) to validate our interpretation of biomarker variability.

## Methods

### Study subjects and data collection

**Figure 1:**
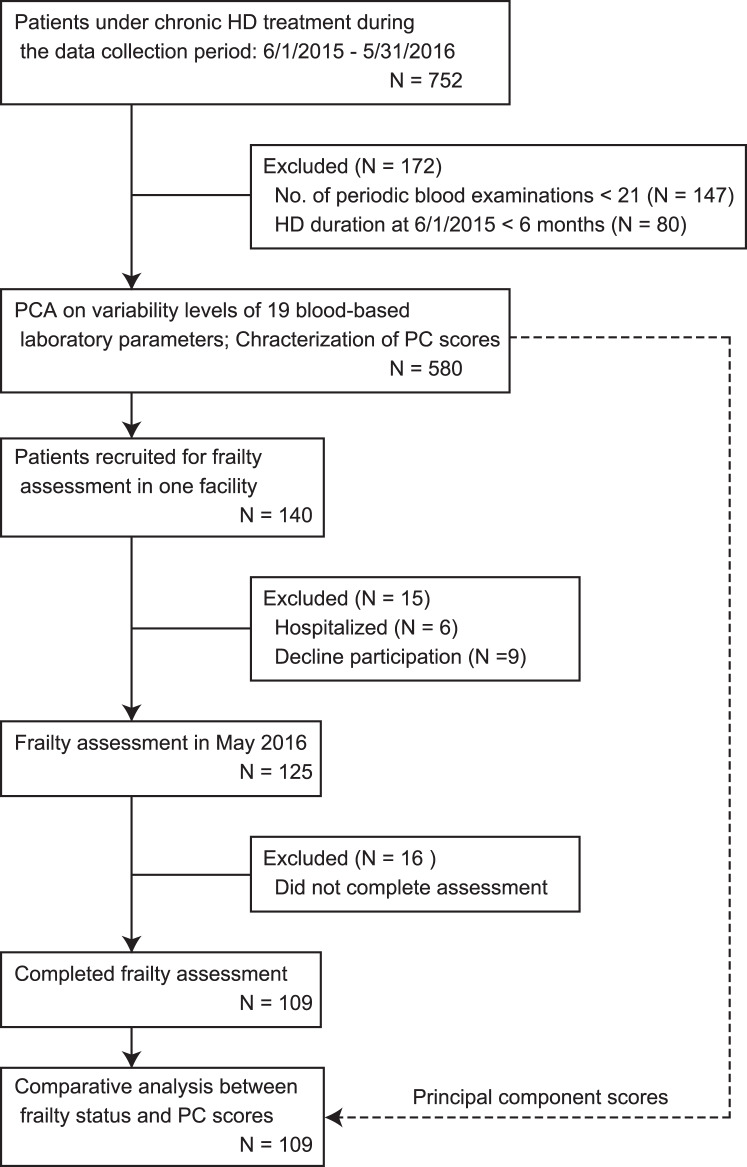
Patient selection and study flow.

A total of 752 patients underwent maintenance HD during the one-year data collection period (from June 2015 until May 2016) at any of 4 affiliated HD facilities. These facilities are located within 6 km of each other and provided equivalent dialysis treatment. To reduce the influence of highly fluctuating parameter values during the HD initiation phase^6^, patients who had been receiving HD for less than 6 months as of the start of the data collection period (namely, those who had started HD treatment later than December 1, 2014) were excluded from this study. We further excluded patients who had completed fewer than 21 of the 24 regular blood examinations scheduled during the data collection period. Finally, we enrolled the remaining 580 patients (Fig. 1).

At each of the 4 participating facilities, 19 blood parameters were regularly examined for all the HD patients. The levels of white blood cells (WBC), hemoglobin (Hb), platelets (Plat), albumin (Alb), blood urea nitrogen (BUN), creatinine (Cr), potassium (K), uncorrected calcium (Ca), and phosphate (P) were measured twice monthly, whereas those of total protein (TP), uric acid (UA), sodium (Na), and chloride (Cl) were measured monthly. Aspartic aminotransferase (AST), alanine aminotransferase (ALT), lactate dehydrogenase (LDH), alkaline phosphatase (ALP), LDL-cholesterol (LDL), and HDL-cholesterol (HDL) levels were measured every 2 months. Among the enrolled patients, 231 had been diagnosed as having diabetes and received additional monthly measurements of their glycated albumin (GA) level. For the regular blood examinations, pre-dialysis blood samples were drawn prior to the first HD treatment of the week and were analyzed at a single external laboratory.

Using the above-mentioned measurements (6–24 measurement points for each parameter), we calculated the mean values and the coefficients of variation (CV = population standard deviation/mean) for each patient^12^. The yearly mean of parameter X (abbreviated as X-M) was regarded as the overall level, and the CV value (X-CV) was used to estimate variability. The CV values were log 10 transformed to normalize the distribution and were expressed as log X-CV or X-LCV. There were no missing data for these M and CV values in the enrolled patients.

### Frailty assessment

Of the 580 enrolled patients, 140 patients who were receiving HD treatment at one facility in May 2016 were recruited for the frailty assessment. At the time of frailty survey, 6 patients were in hospital, and 9 patients declined participation. The remaining 125 patients participated in the assessment, but 16 of them did not complete it. For the final 109 patients without missing data, their frailty status was analyzed relative to the PC scores obtained from a PCA of 580 patients (Fig. 1).

Frailty was defined according to the Japanese version of the Cardiovascular Health Study criteria (J-CHS criteria)^13–15^ as the presence of three or more of five clinical characteristics: (a) weight loss—losing more than 2 kg in the past 6 months; (b) walking speed—less than 1.0 m/second; (c) grip strength—less than 26 kg for men, and less than 18 kg for women; (d) exhaustion—yes in response to the question: “Have you felt tired without any reason in the last 2 weeks?”; and (e) physical activity—no for both of the questions: “Do you perform light exercise or gymnastics?” and “Do you play sports regularly?”.

This study protocol was approved by the institutional ethics committee of Hakuyukai Medical Corporation (approval number: 27–006) and was performed in accordance with the provision of the Declaration of Helsinki. Informed consent for the use of the patients’ clinical records was obtained from all the patients in 2015; the physical and subjective assessments for frailty were conducted in May 2016 after obtaining written informed consent.

### Statistical analysis

Bivariate correlations between continuous variables were assessed using the Spearman or Pearson correlation coefficients according to the distribution of the variables. To compare values between two groups, *P* values were calculated using the Welch t-test or the Fisher exact test, as appropriate. For correlations among the LCVs of the laboratory parameters, we did not adjust the *P* values for multiple comparisons because almost all the LCVs were clearly correlated with one another (see Results). In this situation, such *P* value adjustments merely increase false-negative reporting, rather than decreasing false-positive reporting^16^.

PCA is a linear transformation technique used for feature extraction, dimensionality reduction, and noise reduction. It converts a set of observed variables into a set of orthogonal variables called principal components (PCs) by rotating the coordinate system. PC scores represent the values of the PCs. In this study, PCA was performed on the 19 LCVs after they were standardized to have a mean of zero and a standard deviation (SD) of one. The scores for the 19 PCs were computed for each subject and were used for further analyses.

The associations between the frailty status and the explanatory variables were examined using univariate and multivariate logistic regression models. In these analyses, the frailty status was binarized by combining pre-frail and non-frail patients as a “not-frail” group^17–19^, and the odds ratio for a frail status relative to a not-frail status was estimated. The discriminatory ability of each model was evaluated by computing the area under the receiver operating characteristic curve (AUC = c-statistic) and the accuracy.

All the analyses were performed in R.3.5.0 (R Core Team, 2018) using the corrplot, binomTools, pscl, ROCR, pROC and caret packages. The results were basically expressed as the mean ± SD, and a *P* value <0.05 was considered significant.

## Results

### Patient characteristics

The demographic and laboratory data for the 580 patients who were enrolled in the PCA are shown in Table 1. The patient age was 65.6 ± 12.3 years (range = 25.3–93.0 years), and most of them had received long-term hemodialysis treatment (interquartile range = 4.9–15.5 years). The proportions of female and diabetic patients were 32.1% and 37.8%, respectively.

**Table 1:** Patient characteristics.

	Total	Non-diabetic	Diabetic
Number of patients	580	361	219
Male/Female (n)	394/186	233/128	161/58*
Age (years)	65.6 ± 12.3	65.0 ± 12.8	66.7 ± 11.3
HD duration (years)	11.3 ± 8.4	13.4 ± 9.1	7.8 ± 5.4***
WBC-M (/µl)	6120 ± 1648	5908 ± 1568	6469 ± 1720***
Hb-M (g/dL)	11.1 ± 0.8	11.1 ± 0.8	11.1 ± 0.7
Plat-M (10^4^/µl)	19.1 ± 5.8	19.1 ± 5.5	19.1 ± 6.3
TP-M (g/dL)	6.5 ± 0.4	6.5 ± 0.4	6.5 ± 0.5
Alb-M (g/dL)	3.6 ± 0.3	3.6 ± 0.3	3.6 ± 0.3
AST-M (IU/L)	14.6 ± 7.5	15.0 ± 8.7	14.0 ± 4.8
ALT-M (IU/L)	11.5 ± 5.7	11.6 ± 6.0	11.4 ± 5.2
LDH-M (IU/L)	179 ± 39	179 ± 40	179 ± 33
ALP-M (IU/L)	263 ± 138	270 ± 151	252 ± 113
BUN-M (mg/dL)	64.3 ± 12.2	65.6 ± 11.6	62.3 ± 12.9**
Cr-M (mg/dL)	11.4 ± 2.6	11.8 ± 2.6	10.8 ± 2.5***
UA-M (mg/dL)	7.4 ± 1.2	7.5 ± 1.2	7.1 ± 1.1***
Na-M (mmol/L)	138.8 ± 2.3	139.1 ± 2.2	138.4 ± 2.4**
K-M (mmol/L)	5.0 ± 0.6	5.1 ± 0.6	4.9 ± 0.7**
Cl-M (mmol/L)	105 ± 3.0	105.2 ± 3.0	104.6 ± 2.9*
Ca-M (mg/dL)	8.8 ± 0.4	8.8 ± 0.4	8.8 ± 0.4
P-M (mg/dL)	5.4 ± 1.0	5.5 ± 1.0	5.4 ± 0.9
LDL-M (mg/dL)	84.2 ± 26.7	87.4 ± 26.8	79.0 ± 25.7***
HDL-M (mg/dL)	46.0 ± 13.5	47.7 ± 14.0	43.2 ± 12.3***
GA-M (%)			20.5 ± 4.4
WBC-LCV	−0.91 ± 0.14	−0.92 ± 0.14	−0.89 ± 0.15*
Hb-LCV	−1.27 ± 0.17	−1.28 ± 0.17	−1.25 ± 0.15*
Plat-LCV	−1.02 ± 0.16	−1.03 ± 0.16	−1.02 ± 0.15
TP-LCV	−1.54 ± 0.13	−1.53 ± 0.13	−1.54 ± 0.13
Alb-LCV	−1.39 ± 0.13	−1.40 ± 0.13	−1.38 ± 0.13
ALT-LCV	−0.86 ± 0.28	−0.86 ± 0.29	−0.84 ± 0.27
AST-LCV	−0.77 ± 0.25	−0.78 ± 0.25	−0.75 ± 0.25
LDH-LCV	−1.18 ± 0.21	−1.19 ± 0.21	−1.18 ± 0.20
ALP-LCV	−1.01 ± 0.23	−1.02 ± 0.22	−0.99 ± 0.23
BUN-LCV	−0.92 ± 0.13	−0.94 ± 0.13	−0.89 ± 0.13***
Cr-LCV	−1.27 ± 0.17	−1.30 ± 0.17	−1.22 ± 0.17***
UA-LCV	−1.12 ± 0.16	−1.13 ± 0.15	−1.10 ± 0.18*
Na-LCV	−1.96 ± 0.14	−1.97 ± 0.13	−1.95 ± 0.15*
K-LCV	−1.13 ± 0.15	−1.15 ± 0.14	−1.09 ± 0.16***
Cl-LCV	−1.78 ± 0.14	−1.80 ± 0.14	−1.75 ± 0.14***
Ca-LCV	−1.46 ± 0.17	−1.46 ± 0.17	−1.46 ± 0.16
P-LCV	−0.83 ± 0.13	−0.84 ± 0.13	−0.80 ± 0.13***
LDL-LCV	−1.08 ± 0.21	−1.11 ± 0.19	−1.03 ± 0.23***
HDL-LCV	−1.17 ± 0.19	−1.20 ± 0.19	−1.12 ± 0.18***
GA-LCV			−1.34 ± 0.25

X-M and X-LCV denote yearly mean and log 10-transformed coefficient of variation of parameter X, respectively. Data for diabetic and non-diabetic groups were compared by Fisher’s exact test or Welch’s t test. ***P < 0.001, **P < 0.01, *P < 0.05.

As the diabetic status in HD patients has a strong influence on health conditions, prognosis, and the development of frailty^20,21^, the patient characteristics were also presented separately for diabetic and non-diabetic patients. Compared with non-diabetic patients, diabetic patients had lower mean levels of BUN, Cr, UA, Na, K, Cl, LDL, and HDL, while they had a higher degree of variability (log CV values) for WBC, Hb, BUN, Cr, UA, Na, K, Cl, P, LDL, and HDL.

### Correlations between variables

**Figure 2:**
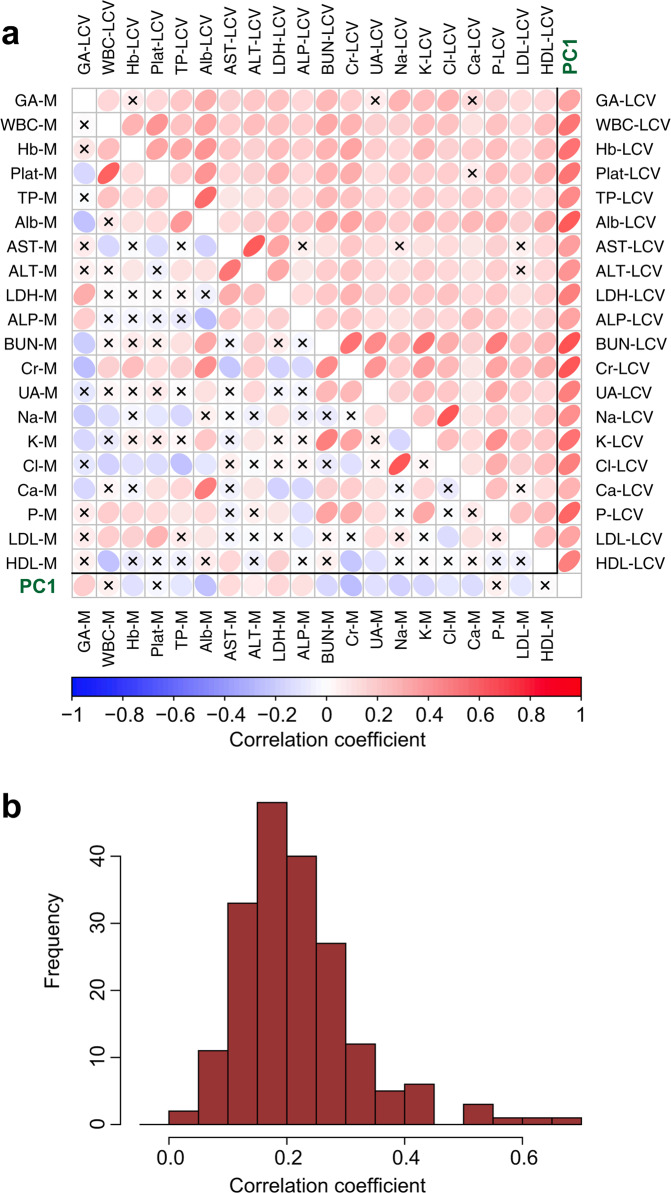
Correlations between variabilities and those between mean levels of 20 laboratory parameters. (**a**) One hundred and ninety pairwise correlation coefficients between 20 LCVs (=log transformed coefficients of variation) were excerpted from Supplementary Table S1 and are shown as ellipses in the upper triangle of the heatmap. Similarly, the correlation coefficients between 20 Ms (=yearly means) are shown in the lower triangle. The sign and strength of the correlation coefficient is displayed by the color, and insignificant correlations (*P* > 0.05) are marked with an **×** (see Methods for details). Additionally, first principal component score (PC1 score) is obtained from the PCA of 19 LCVs (all LCVs except GA-LCV), and the correlations between the 20 LCVs and the PC1 are shown in the rightmost column of the heatmap. The correlations between the 20 Ms and the PC1 are also shown in the bottom row. The actual values of the correlation coefficients are shown in Table 2. (**b**) Distribution of the correlation coefficients between LCVs displayed as a histogram.

The pair-wise correlations among demographics, the mean levels of the laboratory parameters (M), and their variability (LCV) were computed; the entire correlation matrix (44 by 44) is provided as Supplementary Table S1. Subsets of this matrix, specifically the correlation matrix for the 20 LCVs and that for the 20 Ms, were combined and are illustrated as a heatmap in Fig. 2a. As shown in the upper triangle of the heatmap, the 190 correlation coefficients for all possible combinations of LCVs were positive. The coefficient values were generally modest (Fig. 2b), but 181 of them (95.3%) were statistically significant. In sharp contrast, the sign and the strength of the correlation between different Ms varied according to the combination (see lower triangle of Fig. 2a).

### PCA

As GA-LCV data were only available for the diabetic patients, the remaining 19 LCVs were used to build the PCA model. The first 5 principal components showed corresponding eigenvalues of more than 1.0, and the PC1 had an eigenvalue of 5.26 and accounted for 27.7% of the total variance. The corresponding values for PC2 were much lower (1.58 and 8.3%, respectively). The variable loadings for PC1 were all positive (range = 0.140 to 0.305); thus, PC1 was regarded as a cumulative index of variability. Likewise, as shown in the left half of Table 2, the correlation coefficients between the PC1 score and the original 19 LCVs (i.e., component loadings for PC1) were all positive (range = 0.321 to 0.701). These results indicate that PC1 represents principal information shared by all the LCVs. The correlations between PC1 and other variables (right half of Table 2) revealed a consistent relationship with multiple prognostic factors in HD patients. The PC1 score was negatively correlated with Alb-M, Cr-M, K-M, Na-M, BUN-M, Ca-M, Hb-M, and LDL-M, etc. On the other hand, it was positively correlated with GA-LCV, GA-M, AST-M, LDH-M, diabetic status, ALP-M, age, and ALT-M. These correlations are also displayed as a heatmap in Fig. 2a.

**Table 2:** Correlations between PC1 score and study variables.

Variables	r	*P* value	Variables	r	*P* value
Ca-LCV	0.321	**0.000**	Alb-M	−0.278	**0.000**
ALP-LCV	0.364	**0.000**	Cr-M	−0.254	**0.000**
LDL-LCV	0.375	**0.000**	K-M	−0.173	**0.000**
AST-LCV	0.380	**0.000**	Na-M	−0.171	**0.000**
ALT-LCV	0.430	**0.000**	BUN-M	−0.162	**0.000**
Na-LCV	0.449	**0.000**	Ca-M	−0.138	**0.001**
TP-LCV	0.485	**0.000**	Hb-M	−0.118	**0.005**
UA-LCV	0.490	**0.000**	LDL-M	−0.104	**0.013**
LDH-LCV	0.493	**0.000**	Cl-M	−0.102	**0.014**
HDL-LCV	0.506	**0.000**	UA-M	−0.102	**0.014**
Cl-LCV	0.520	**0.000**	TP-M	−0.098	**0.018**
WBC-LCV	0.561	**0.000**	HD duration	−0.081	0.051
Hb-LCV	0.566	**0.000**	Plat-M	−0.031	0.450
K-LCV	0.584	**0.000**	HDL-M	−0.015	0.712
Plat-LCV	0.590	**0.000**	Female (vs. male)	−0.006	0.890
P-LCV	0.614	**0.000**	WBC-M	0.052	0.212
Cr-LCV	0.663	**0.000**	P-M	0.073	0.080
Alb-LCV	0.693	**0.000**	ALT-M	0.101	**0.015**
BUN-LCV	0.701	**0.000**	Age	0.137	**0.001**
			ALP-M	0.137	**0.001**
			DM (vs. non-DM)	0.195	**0.000**
			LDH-M	0.200	**0.000**
			AST-M	0.221	**0.000**
			GA-M^a^	0.240	**0.000**
			GA-LCV^a^	0.366	**0.000**

DM = diabetes mellitus. The correlation coefficients (r) between PC1 and the original variables of the PCA, namely the 19 LCVs, are displayed on the left, and the r for other variables (19 Ms, GA-LCV, DM, HD duration, and gender) are displayed on the right. For dichotomous variables, r represents the point-biserial correlation coefficient. Variables are arranged in the order of their r values from lowest to highest. Boldface indicates a statistical significance at *P* < 0.05. ^a^For GA-M ^a^nd GA-LCV, the coefficients were calculated for diabetic subjects only.

### Frailty

The relationships between frailty status and demographic/laboratory data were analyzed for 109 of the 580 patients. Among them, 23 (21.1%) and 55 (50.5%) patients were classified as frail and pre-frail, respectively. When the patients were re-classified into two categories, namely frail and not-frail (pre-frail + non-frail), several characteristics differed between the 2 groups (Table 3). The frail patients were generally older, had a longer duration of HD, and had lower Alb and Cr levels and higher LDH and ALP levels. Of note, the frail patients exhibited elevated LCV levels for all the parameters except for UA, though the differences were statistically significant only for Hb-LCV, Plat-LCV, Alb-LCV, and LDH-LCV. The PC scores derived from a PCA of 580 patients were also compared. Of the 19 PC scores, the PC1 score was significantly higher in frail patients than in not-frail patients, but the other PC scores were not significantly different between the 2 groups (Table 3).

**Table 3:** Patient characteristics according to frailty status.

Variables	Not-frail	Frail	SMD	*P* value	Variables	Not-frail	Frail	SMD	*P* value
Demographics					Principal component score				
Age	61.7 ± 11.6	69.8 ± 7.4		**0.000**	PC1	-0.66 ± 1.76	0.74 ± 2.52	0.61	**0.019**
Female (%)	29.1	30.4		0.902	PC2	0.19 ± 1.16	-0.18 ± 1.21	-0.29	0.199
HD duration	9.9 ± 8.2	17.9 ± 10.5		**0.002**	PC3	0.10 ± 0.99	0.34 ± 1.24	0.21	0.388
DM (%)	30.2	43.5		0.265	PC4	0.00 ± 1.06	-0.12 ± 0.90	-0.11	0.586
Biomarker level					Biomarker variability				
LDH-M	175 ± 27	202 ± 55	0.68	**0.034**	Alb-LCV	-1.41 ± 0.09	-1.33 ± 0.15	0.63	**0.021**
ALP-M	236 ± 76	298 ± 123	0.45	**0.029**	LDH-LCV	-1.23 ± 0.20	-1.10 ± 0.27	0.62	**0.042**
Na-M	139.0 ± 2.0	140.0 ± 2.0	0.24	0.243	Hb-LCV	-1.30 ± 0.16	-1.21 ± 0.16	0.53	**0.025**
AST-M	14.3 ± 4.6	15.0 ± 3.9	0.09	0.471	LDL-LCV	-1.12 ± 0.19	-1.02 ± 0.24	0.48	0.070
Cl-M	103.0 ± 2.7	103.0 ± 2.4	0.08	0.681	Plat-LCV	-1.04 ± 0.14	-0.97 ± 0.14	0.48	**0.031**
K-M	4.94 ± 0.57	4.96 ± 0.64	0.03	0.904	Ca-LCV	-1.46 ± 0.18	-1.39 ± 0.18	0.43	0.089
UA-M	7.45 ± 1.12	7.46 ± 0.77	0.01	0.960	ALT-LCV	-0.84 ± 0.25	-0.73 ± 0.28	0.42	0.113
ALT-M	11.6 ± 3.9	11.3 ± 4.9	-0.04	0.840	P-LCV	-0.83 ± 0.11	-0.77 ± 0.14	0.39	0.100
Plat-M	19.4 ± 5.2	18.9 ± 7.0	-0.10	0.714	BUN-LCV	-0.96 ± 0.11	-0.91 ± 0.11	0.39	0.062
WBC-M	6030 ± 1790	5860 ± 1770	-0.10	0.684	AST-LCV	-0.91 ± 0.25	-0.82 ± 0.32	0.33	0.210
LDL-M	90.4 ± 25.7	87.3 ± 25.3	-0.12	0.602	Cl-LCV	-1.80 ± 0.14	-1.76 ± 0.12	0.26	0.200
P-M	5.41 ± 0.84	5.18 ± 0.76	-0.25	0.204	TP-LCV	-1.54 ± 0.11	-1.51 ± 0.13	0.25	0.266
HDL-M	49.0 ± 15.7	45.2 ± 14.8	-0.29	0.279	K-LCV	-1.13 ± 0.13	-1.10 ± 0.16	0.22	0.352
Ca-M	8.83 ± 0.47	8.70 ± 0.50	-0.30	0.262	WBC-LCV	-0.95 ± 0.12	-0.92 ± 0.15	0.22	0.361
TP-M	6.56 ± 0.39	6.41 ± 0.38	-0.36	0.101	Cr-LCV	-1.30 ± 0.17	-1.27 ± 0.13	0.20	0.320
BUN-M	64.7 ± 13.1	60.2 ± 11.2	-0.37	0.107	HDL-LCV	-1.20 ± 0.19	-1.17 ± 0.16	0.15	0.471
Cr-M	11.80 ± 2.56	10.60 ± 1.78	-0.46	**0.014**	Na-LCV	-1.99 ± 0.10	-1.98 ± 0.14	0.04	0.863
Hb-M	11.10 ± 0.65	10.70 ± 1.01	-0.53	0.081	ALP-LCV	-1.03 ± 0.22	-1.03 ± 0.28	0.00	0.995
Alb-M	3.66 ± 0.27	3.38 ± 0.31	-0.89	**0.000**	UA-LCV	-1.13 ± 0.19	-1.14 ± 0.20	-0.03	0.925

The patient characteristics of the not-frail group (n = 86) and the frail group (n = 23) were compared using the Welch t-test or the Fisher exact test. The units of the variables are the same as those used in Table 1. PC1-4 = first to fourth principal components, SMD = standardized mean difference between the 2 groups. A positive SMD denotes a higher average value for the frail group. Boldface indicates statistical significance at *P* < 0.05. The values for the 19 Ms (left side) and those for the 19 LCVs (right side) are arranged in order of their respective SMD values. The values for PC5 or lower ranked PCs were omitted.

**Figure 3:**
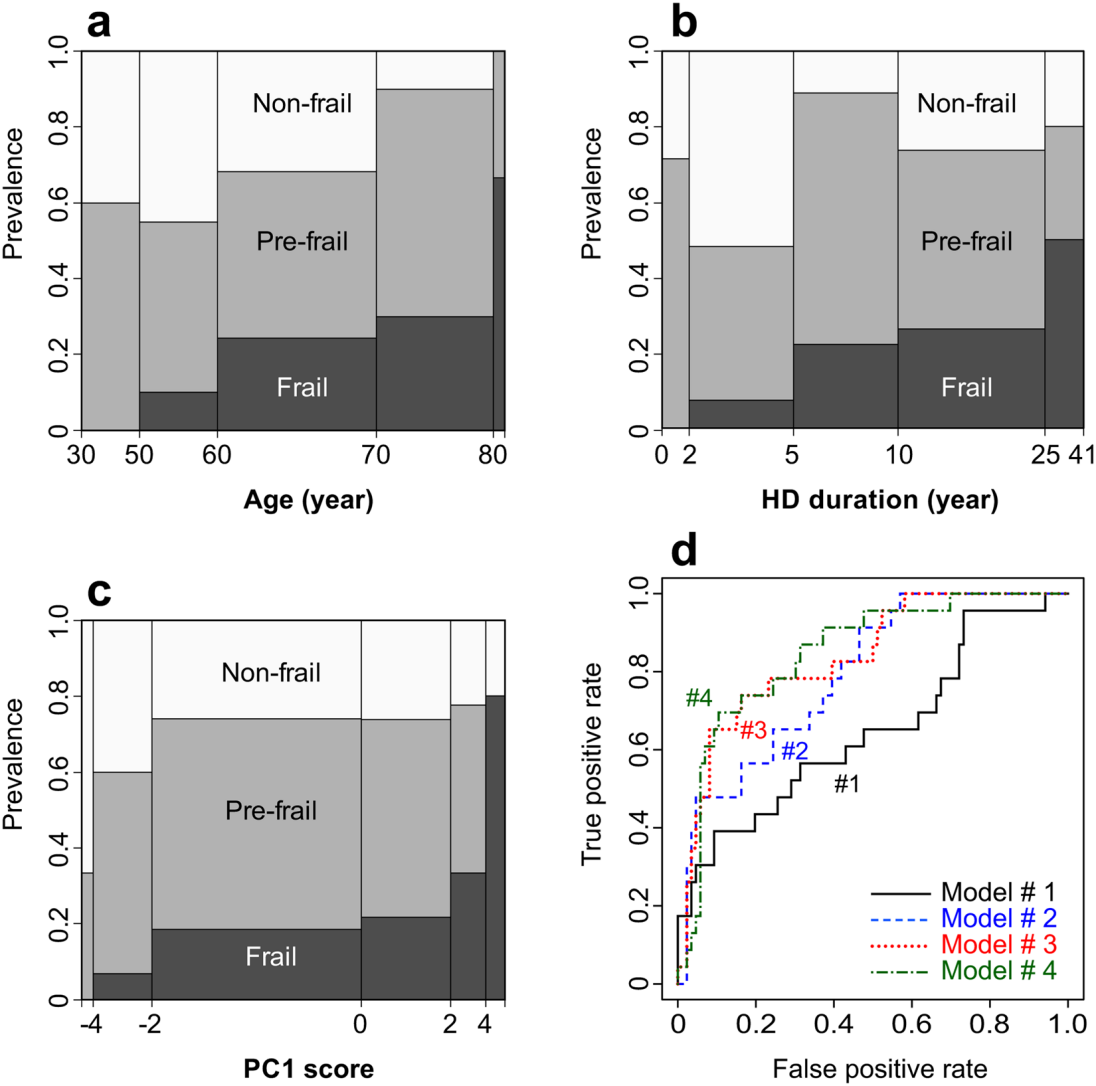
Prevalence of frailty and performance of predictive models. (**a**–**c**) Subjects were stratified according to age, HD duration, or PC1 score, and their frailty levels were displayed as mosaic plots. The areas of the tiles are proportional to the numbers of subjects. (**d**) Receiver operating characteristic curves of combined classifiers for frailty. Models #1 − #4 correspond to those in Table 4.

As shown in Fig. 3, the prevalence of frailty tended to increase with increasing age, HD duration, and PC1 score, though there was no significant correlation between these 3 variables in the examined subjects. The discriminatory power of the PC1 score for frailty was analyzed using logistic regression models and receiver operating characteristic curves (Table 4 and Fig. 3d). In these models, mean levels of laboratory parameters (Ms) are not included in the covariates because they are broadly correlated with each other and with PC1 and can cause multicollinearity problems. The PC1 score, age, and HD duration were independently associated with frailty in these univariate and multivariate models, and the PC1 score had odds ratios (OR) of 2.20–2.42 (per 1 SD) for frailty.

**Table 4:** Logistic regression models for frailty.

	Predictor	OR (95% CI)	*P* value	pR^2^	AIC	AUC (95% CI)	Accuracy	Sensitivity	Specificity
Univariate model									
#1	PC1 (per SD)	2.20 (1.26–3.81)	**0.005**	0.08	107.8	0.649 (0.512–0.787)	0.817	0.174	0.988
Multivariate models									
#2	Age	1.09 (1.02–1.16)	**0.006**						
	HD duration	1.09 (1.03–1.15)	**0.002**	0.18	96.5	0.795 (0.700–0.891)	0.835	0.348	0.965
#3	PC1	2.42 (1.30–4.53)	**0.006**						
	Age	1.10 (1.03–1.17)	**0.004**						
	HD duration	1.09 (1.03–1.15)	**0.003**	0.27	90.0	0.838 (0.748–0.928)	0.835	0.478	0.930
#4	PC1	2.31 (1.22–4.36)	**0.010**						
	Age	1.09 (1.02–1.17)	**0.008**						
	HD duration	1.11 (1.04–1.19)	**0.001**						
	DM	2.74 (0.78–9.60)	0.115	0.29	89.5	0.849 (0.764–0.934)	0.862	0.565	0.942
#5	PC1	2.34 (1.21–4.52)	**0.011**						
	Age	1.09 (1.02–1.17)	**0.008**						
	HD duration	1.11 (1.04–1.19)	**0.001**						
	Female	1.14 (0.32–4.05)	0.837						
	DM	2.76 (0.79–9.72)	0.113	0.29	91.4	0.854 (0.770–0.938)	0.862	0.565	0.942

OR = odds ratio, *P* value = *P* value of Wald’s test, pR^2^ = McFadden’s pseudo R^2^, AIC = Akaike’s Information Criterion, AUC = area under the curve of receiver operating characteristics. *P* value printed in boldface indicates *P* < 0.05. Models #1 − #4 correspond to those in Fig. 3d.

The PC1 score alone was not sufficient to determine the frailty status (AUC = 0.649, accuracy = 0.817, model #1 in Table 4 and Fig. 3d). However, as shown by the comparison between model #2 and model #3, its addition to the explanatory variables significantly improved the fit of the model (*P* = 0.0037 for likelihood ratio test). The lower ranked PCs (PC2 − PC19) had no significant association with frailty status, and their further addition to these models did not improve the performance. A multivariate model (#4 in Table 4 and Fig. 3d) containing 4 variables (PC1 score, age, HD duration, and diabetic status) had the smallest AIC and an AUC of 0.849, indicating good discrimination. At the probability cutoff value of 0.5, this model showed 0.565 sensitivity, 0.942 specificity, and 0.862 accuracy. To obtain an unbiased estimate of the AUC, a 10-fold cross validation was repeated 100 times. With this resampling method, the averaged AUC of model #4 was 0.824.

## Discussion

In our previous survival study, we observed widespread positive correlations between LCVs in the baseline data which had been recorded during the year of 2002^4^. The dataset analyzed in the present study was collected more than 12 years thereafter. Nevertheless, the correlation matrices derived from both datasets showed very similar patterns and levels (compare Table 5 in Ref. ^4^ and Supplementary Table S1), indicating their nearly constant correlation structure. Given that a high LCV level reflects dysfunction of the corresponding physiological regulatory system, the correlation coefficient between two different LCVs can represent the strength of the interaction (coupling) between two different physiological systems^22^. More specifically, the robust correlation (r > 0.5) between certain pairs of LCVs (Na-LCV/Cl-LCV, BUN-LCV/Cr-LCV, Alb-LCV/TP-LCV, and BUN-LCV/K-LCV, see Supplementary Table S1) indicates the proximity of the regulatory mechanisms of these paired parameters. Taking this idea one step further, the ubiquitous positive correlations among the LCVs imply an interconnected structure (i.e., network) of homeostatic regulation and suggest that its overall dysfunction can be estimated by PC1 of the LCVs^22,23^.

Changes in physiological variability in relation to aging and health can be viewed through two lenses that produce contradictory results. On the one hand, the “loss of complexity” paradigm suggests that high variability is a sign of a system that is able to adjust appropriately, and that loss of variability is a sign of loss of appropriate complexity (ref. ^23^ and others by Lipsitz and Goldberger). On the other hand, the “critical transitions” paradigm suggests that high variability is a marker of an impending critical transition in system state, usually undesirable in a health context^24,25^. Perhaps as a bridge between these two, Fossion *et al*. suggest that physiological variables can be divided into regulated variables (those that are kept stable, i.e., targets of homeostasis) and physiological responses (buffers that adjust in order to keep regulated variables stable)^8^. This is highly concordant with sub-cellular regulation as demonstrated by Nijhout *et al*.^26^. However, our results would seem to provide unmitigated support for the critical transition framework, with generalized increases in variability observed in frailty across all biomarkers, regardless of whether they might be thought to be regulated or responses. Why this is remains to be explored, though some publications have questioned whether observed changes in heart rate variability that motivated the “loss of complexity” framework are indeed reproducible^27^.

Glycemic variability (GV) has been assessed by repeated measurements of blood glucose, hemoglobin A1c, or GA levels with various sampling intervals. Despite the different definitions of GV, studies have generally reported associations between a high GV and adverse patient outcomes^12,28,29^. GV is an active topic of clinical medicine, and we were interested in the similarity between GV and other LCVs. In chronic HD treatment, the GA value has been recognized as a superior index of glycemic control^30^, and the dataset used in this study contained periodic GA values; therefore, both the GA-M and the GA-LCV were included in the analysis. In the diabetic patients, the GA-LCV was significantly correlated with 16 of the 19 LCVs and, like most of the other LCVs, exhibited negative correlations with Alb-M, Cr-M, and Na-M, all three of which are solid prognostic predictors (Fig. 2a and Supplementary Table S1).

While GA values were not available for non-diabetic subjects, we think that these correlations likely exist in the entire subjects for the following reasons: (a) GA-LCV was strongly correlated with GA-M in the diabetic patients (r = 0.54, Supplementary Table S1), (b) the diabetic patients had relatively high levels for the majority of LCVs (Table 1), and (c) the GA-M and GA-LCV levels of non-diabetic patients should be lower than those of diabetic patients. Accordingly, GA-LCV also seems to share common characteristics and significance with other LCVs. That is, the regulation of blood glucose is not independent from that of other blood components. Until now, a high GV has been discussed only in the context of diabetic complications. However, as a high GA-LCV level, along with a high GA-M level, is largely accompanied by wide fluctuations in other parameters, at least in HD patients, it could also be a manifestation of diverse (not necessarily diabetes-related) types of organ/tissue damages and accompanying physiological dysregulation. This interpretation is compatible with the presence of impaired glucose tolerance in patients with various chronic diseases^31–33^ as well as frail elderly^34^. Furthermore, it explains why dysglycemia in critically ill patients is associated with a high mortality and why strict glycemic control has a minimal effect on their prognosis^35^.

Most of the operational criteria for frailty are based on aggregate scores of survey items, which were selected empirically to capture physical, mental, and social well-being. Frailty assessments can thus be time-consuming and require the cooperation of the subjects, but they still entail some uncertainty because of the lack of an objective definition. Accordingly, biomarkers that complement frailty assessments have been sought and proposed^36^. Some of the LCVs examined in the present study are candidates for such biomarkers, since their values were often associated with aspects of frailty^4^. When comparing the levels of the 19 LCVs between frail and not-frail groups, the frail group actually showed elevated mean LCV values for almost all the parameters, though the difference was moderately significant for only 4 parameters (Table 3). In comparison, the PC1 score showed a more significant difference than each of the LCVs, indicating that the former is a reliable marker for estimating frailty. In line with this result, a multivariate logistic regression model containing only 4 predictors (PC1, age, HD duration, and diabetic status) reasonably discriminated the frailty status without the use of physical performance tests or questionnaires. While the PC1 score alone is a moderate predictor of frailty, the information it contains appears to be largely independent of age and other covariates in the subjects. Furthermore, the PC1 score was associated with many parameters in a manner that was consistent with their prognostic significance in HD patients (see the right side of Table 2). They include diabetes (or high GA-M), high GV, and low serum levels of Alb, Cr, K, Na, BUN, Hb, LDL-cholesterol, and so on. As deviated levels of these parameters are closely linked to frailty, sarcopenia, protein energy wasting, and mortality risk in HD patients^36–41^, our results indicate that the PC1 of LCVs is certainly a marker of adverse health conditions. It is currently unknown why blood levels of each of these parameters have a different (i.e., positive or negative) relationship with prognosis. This diversity probably reflects the uniqueness of each regulatory system, and we speculate that the common basis of various poor prognostic factors might be their close association with a high PC1 level, which denotes a diminished homeostatic capacity.

For HD patients, the PC1 score can be calculated from routine blood examinations and can also be used to objectively estimate their frailty status. We believe that the PCA-based frailty estimation is clinically applicable and will be a useful guide in selecting treatment options for patients with co-morbidities.

PCA has some favorable properties in exploring LCVs. Several biomarkers, including P, K, UA, and BUN, reportedly have a U-shaped relationship between their levels and mortality^42–44^. On the other hand, the extent of their variability appeared to have a monotonic effect on the mortality risk in previous studies^4,45–47^. Thus, we can simply apply LCVs to PCA without considering their normal or desirable values. Unlike ordinary multiple regression modelling, PCA is not impeded by multicollinearity, which was moderately but widely present among the LCVs. Moreover, since PCA is a non-supervised analysis, the resultant PC scores represent information inherent to the data and can be used as independent variables in different regression models for various definitions of frailty/sarcopenia as well as mortality. In PCA for variables with the same directional properties, as the number of variables increases, the result seems to be less affected by the number and selection of the variables^48^. Using this same dataset, we also examined a smaller PCA model based on 9 variables, namely LCVs of WBC, Hb, Plat, Alb, BUN, Cr, K, Ca, and P. Although the detailed results were omitted to avoid repetition, the PC1 score was strongly correlated with the score from the original PCA model based on 19 LCVs (r = 0.95) and provided nearly identical results in the logistic regression analyses for frailty. For example, the model corresponding to the original model #4 exhibited a *P* value of 0.008, an AUC of 0.861, and an accuracy of 0.844. This property helps to generate common PC1 scores from multiple datasets, each containing a different set of parameters.

The main limitation of this study is the relatively small sample size of patients who had completed the frailty assessment, which might have reduced the statistical power to detect differences between the frail and not-frail groups. We also excluded patients who were hospitalized at the time of the survey and those who could not respond to the questionnaire. Hence, the subjects who were included in the frailty analysis might have been less frail, compared with the overall subjects in the study. Although these situations may have weakened some of the results, we think that the presently reported conclusions are still very clear and reasonable.

Another limitation is the cross-sectional design. The regression model produced in this study demonstrated that HD patients with an older age, longer HD duration, and greater fluctuations in laboratory data were generally more frail. Considering the ageing/death-associated changes in Alb-LCV^6^, which is robustly correlated with PC1, this model seems to fit well with the expected time-dependent progression of frailty. However, a cross-sectional study addresses only the prevalence of frailty and can suffer from a selection bias and a survivorship bias. To investigate the relationship between PC1 and frailty more accurately, longitudinal data for both variables will need to be analyzed.

Finally, we should mention that this study was based solely on clinical data from HD patients. The reason for this is that the regular and frequent collection of multi-dimensional data is very difficult to achieve in other populations. Consequently, whether similar results are observable in other disease populations is currently unknown.

In summary, we applied a PCA to the levels of variability of 19 blood-based parameters to explore the physiological implications of variability. The 19 LCVs had similar characteristics and shared common information, which could be extracted as PC1. Compared with the original LCVs, the PC1 score was consistently correlated with frailty as well as various other negative health indicators in HD patients. We concluded that the PC1, which is a cumulative index of variability, is a measure of homeostatic dysregulation and can be used to estimate frailty.

## Supplementary information

Supplementary Information.

## Data Availability

All processed data generated during this study are included in this published article and its Supplementary Information, but the raw data cannot be made openly available to protect the confidentiality of personal information and to comply with the terms of the patient’s consent. Requests related to the raw data should be addressed to the corresponding author.
